# Cystitis glandularis: A controversial premalignant lesion

**DOI:** 10.3892/ol.2014.2360

**Published:** 2014-07-18

**Authors:** XIANLIN YI, HAOYUAN LU, YUEXIAN WU, YANG SHEN, QINGGUI MENG, JIWENG CHENG, YONG TANG, FENGXUE WU, RUBIAO OU, SHAOJUN JIANG, XIANZHONG BAI, KEJI XIE

**Affiliations:** 1 Department of Urology, Tumor Hospital of Guangxi Medical University and Guangxi Cancer Research Institute, Nanning, Guangxi 530021, P.R. China; 2 Department of Respiratory Diseases, Nanfang Hospital, Southern Medical University, Guangzhou, Guangdong 510515, P.R. China; 3 Emergency Department, Jingzhou Hospital of Tongji Medical College of Huazhong, Wuhan, Hubei 432020, P.R. China; 4 Department of Urology, Guangzhou First People’s Hospital, Guangzhou Medical University, Guangzhou, Guangdong 510180, P.R. China

**Keywords:** bladder, cystitis, carcinoma

## Abstract

Cystitis glandularis (CG) has been hypothesized as a potential precursor of adenocarcinoma, although this remains controversial. The present study reports data accumulated from 166 cases of cystitis glandularis with follow-up periods ranging between 0.5 and 17 years. The association between intestinal and typical CG and bladder carcinoma was retrospectively evaluated. The patients included in the present study had presented with typical (n=155) or intestinal (n=11) CG between 1994 and 2010. Of those patients, concurrent carcinoma of the bladder was identified in 15 (9.0%) patients, including two cases of squamous cell carcinoma and 1 case of sarcoma. The cases of carcinoma were identified either prior to or concurrently with the diagnosis of CG. Follow-up was available for 9/11 (81.8%) patients with intestinal CG. Nine months following transurethral fulguration, 8/11 (72.7%) patients were in complete remission and 1/11 (9.1%) complained of urgency and dysuria; two patients were lost to follow-up. The follow-up of the patients ranged from 0.7 to 4.5 years (median, 2.67 years; mean, 2.82 years). No evidence of subsequent carcinoma was identified in any of the patients during the follow-up of the intestinal and typical CG groups. In addition, there was no evidence of carcinoma subsequent to CG in either of the typical or intestinal CG groups. The results did not support that CG increases the future risk of malignancy in the short term and repeated cystoscopies over a short period of time are not recommended.

## Introduction

Cystitis glandularis (CG) is an unusual proliferative disorder of the urinary bladder, which is characterized by transitional cells that have undergone glandular metaplasia (1). CG was first described by Morgagni *et al* (2) in 1761, however, the natural history of CG in clinical practice remained unknown until recently (2,3). In 1950, a study by Immergut and Cottler (4) implicated CG in the development of adenocarcinoma of the bladder (4). Since then, CG has occasionally been proposed as a precursor of adenocarcinoma by various studies (5–14). The intestinal subtype of CG and diffuse lesions have been described as premalignant (15–18); however, certain authors have considered CG to be a chronic and quiescent histologic lesion without any clinical significance (19). Furthermore, Smith *et al* (20) found no evidence that CG increased the future risk of malignancy after follow-up for ~2.6 years. Furthermore, Corica *et al* (21) considered that intestinal metaplasia was not a marked risk factor for bladder cancer.

Due to a lack of definitive evidence regarding the potential premalignant nature of CG and the issues associated with determining an accurate prognosis, the present retrospective study demonstrates 166 cases of CG with follow-up periods ranging between 0.5 and 17 years.

## Patients and methods

### Patients with CG

**Figure 1:**
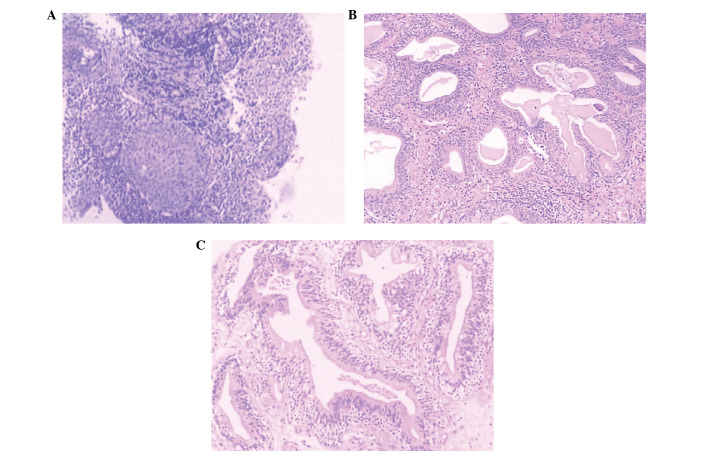
(A and B) Typical cystitis glandularis. Urothelium show reactive changes and underlying proliferation of von Brunn’s nests. (C) Intestinal cystitis glandularis. Sections show the presence of goblet cells and a morphological similiarity to colonic mucosa (stain, trypan blue; magnification, ×100).

A retrospective review of 166 cases of CG between 1994 and 2010 was performed at the Tumor Hospital of Guangxi Medical University (Nanning, China), Nanfang Hospital of Southern Medical University (Guangzhou, China) and Guangzhou First People’s Hospital (Guangzhou, China). All cases were identified as either typical or intestinal type CG by surgical pathology, as reviewed by two pathologists. Clinical information was obtained from the patients’ clinical charts. The patients included in the current study were diagnosed using biopsy or surgical specimens, with the majority of patients possessing data from the two. The association between intestinal and typical CG, and bladder carcinoma was evaluated. Patients provided written informed consent. The glandular structures, which are formed by nests of columnar epithelial cells within the lamina propria of the bladder, were classed into a typical CG group (Fig. 1A and B). The cells of the intestinal CG group exhibited similar glandular structures, however, the simple columnar epithelial cells were replaced by mucin-secreting goblet cells (15–18) (Fig. 1C).

### Statistical analysis

Student’s t-test was used to compare the distribution of age and gender. Statistical analyses were performed using SPSS software (version 13; SPSS, Inc., Chicago, IL, USA). Data are presented as the mean ± standard error of mean. All P-values were two-tailed and P<0.05 was considered to indicate a statistically significant difference.

## Results

A total of 166 patients were identified for inclusion in the present study, who demonstrated typical (n=155) or intestinal (n=11) CG. The patient age ranged from 9 to 86 years and there was no statistically significant difference identified between the two groups with regard to the distribution of age or gender (Table I), although the age range was greater in the typical CG group.

A documented follow-up was available for 114/166 (68.7%) patients and ranged from 0.5 to 17 years (median, 4.22 years; mean, 3.38 years). Six of the 155 (3.9%) patients with typical CG succumbed due to a previous or concurrent cancer.

Follow-up was available for 9/11 (81.8%) patients with intestinal CG. Of these patient, 8/11 (72.7%) were in complete remission following a transurethral fulguration and 1/11 (9.1%) complained of urgency and dysuria 9 months following a transurethral fulguration and intravesical instillation; two patients were lost to follow-up. The follow-up period ranged from 0.7 to 4.5 years (median, 2.67 years; mean, 2.82 years).

All patients with follow-up data in the intestinal and typical CG groups showed no evidence of a subsequent carcinoma. The follow-up procedures included an additional cystoscopy, urine cytology and a clinical examination.

A concurrent carcinoma of the bladder was identified in 15/166 (9.0%) patients. These included two cases of squamous cell carcinoma and one case of sarcoma, all of which were found prior to or concurrently with CG. Other types of concurrent cancer included two cases of cervical, one case of breast and three cases of colon cancer. In one case, CG was identified prior to the presentation of colon cancer.

## Discussion

The natural history of CG in the clinical setting remains unclear. Shaw *et al* (22) first described a case of CG with a gradual transition to adenocarcinoma in 1958. The patient had an extensive history of chronic urinary tract infection that eventually progressed to adenocarcinoma. The biopsy sampling was not conducted in this case due to a large mass that was present at the time of diagnosis (21). Hereafter, two cases of a conversion from CG to adenocarcinoma have been reported (7,23) and it has been proposed that these findings support that CG should be considered as a precancerous lesion (7). By contrast, Ito *et al* (24) reported the characteristic development of CG in 40–92% of apparently healthy bladders in a review of 125 autopsy cases. In these cases, no evidence of malignancy had been found (24). Furthermore, Smith *et al* (20) and Corica *et al* (21) found no evidence that CG or intestinal metaplasia increases the future risk of malignancy.

Although CG has been implicated as a premalignant condition in >16 case reports (20), in 10/16 the evidence was based on the high incidence of a coexistence of CG and adenocarcinoma (20,25). The present study found no evidence of carcinoma subsequent to typical or intestinal CG with a 0.5 to 17-year follow-up. The results did not support that CG increases the future risk of malignancy, however, this was only evaluated in the short term. In addition, repeated cystoscopies over a short period are not recommended (20).

In conclusion, a limitation of the present study was that the follow-up period may not have been long enough. The progression of CG to cancer may be a long-term process, therefore, definitive evidence is lacking from the present study and a longer follow-up period is required (1). Currently, a therapeutic recommendation for CG cannot be proposed, due to the lack of previous cases reported and due to uncertainty regarding the etiopathogenesis (26). Therefore, a large-scale, multi-center follow-up study is required in order to facilitate the accurate determination of a prognosis for patients with CG.

## Figures and Tables

**Table I:** Age and gender distribution of intestinal and typical CG.

Diagnosis	Male (n)	Female (n)	t-value	P-value	Minimum age (years)	Maximum age (years)	Mean age (years)	t-value	P-value
CG			1.045	0.177				0.954	0.341
Typical	73	82			9	86	51.1±16.1		
Intestinal	8	3			34	68	46.4±12.7		

CG, cystitis glandularis. Data are presented as the mean ± standard deviation.
